# The Natural Compound Neobractatin Induces Cell Cycle Arrest by Regulating E2F1 and Gadd45α

**DOI:** 10.3389/fonc.2019.00654

**Published:** 2019-07-17

**Authors:** Zhaoqing Zheng, Man Wu, Juan Zhang, Wenwei Fu, Naihan Xu, Yuanzhi Lao, Lan Lin, Hongxi Xu

**Affiliations:** ^1^School of Pharmacy, Shanghai University of Traditional Chinese Medicine, Shanghai, China; ^2^Engineering Research Center of Shanghai Colleges for TCM New Drug Discovery, Shanghai, China; ^3^Key Lab in Health Science and Technology, Division of Life Science, Graduate School at Shenzhen, Tsinghua University, Shenzhen, China; ^4^Perelman School of Medicine, The Children's Hospital of Philadelphia, University of Pennsylvania, Philadelphia, PA, United States

**Keywords:** neobractatin, cell cycle, E2F1, GADD45α, RNA-Seq, GSEA

## Abstract

The complexity and multi-target feature of natural compounds have made it difficult to elucidate their mechanism of action (MoA), which hindered the development of lead anticancer compounds to some extent. In this study, we applied RNA-Seq and GSEA transcriptome analysis to rapidly and efficiently evaluate the anticancer mechanisms of neobractatin (NBT), a caged prenylxanthone isolated from the Chinese herb *Garcinia bracteata*. We found that NBT exerted anti-proliferative effect on various cancer cells and caused both G1/S and G2/M arrest in synchronized cancer cells through its effects on the expression of E2F1 and GADD45α. The *in vivo* animal study further suggested that NBT could reduce tumor burden in HeLa xenograft model with no apparent toxicity. By demonstrating the biological effect of NBT, we provided evidences for further investigations of this novel natural compound with anticancer potential.

## Introduction

Active compounds from traditional Chinese medicine (TCM) have long been recognized as valuable sources of anticancer drugs. Camptothecin, for instance, is one of approved anticancer drugs deprived from TCM (1). The *Garcinia* species of Chinese herb has been studied for nearly 80 years. Many compounds isolated from these plants, including benzophenones, caged xanthones, and polycyclic polyprenylated acylphloroglucinol (PPAPs), have been shown to have anticancer potential (2). Gambogic acid, for example, is a promising anticancer agent that underwent phase II clinical trials in China in patients with non–small-cell lung, colon, and renal cancers (3). Our previous study showed that neobractatin (NBT), a caged prenylxanthone isolated from *Garcinia bracteata* C. Y. Wu ex Y. H. L, could strongly induce apoptosis, as well as inhibit autophagic flux and cell proliferation in both A549 and HeLa cell lines (4). However, the detailed mechanism by which NBT exerts its antiproliferative effect on cancer cells remains largely unknown.

Similar to other natural compounds, the key challenge to identify the mechanism of the anticancer activity of NBT lies on its complexity that may affect multiple cellular targets. The application of RNA-Seq, which has revolutionized our ability to analyze eukaryotic transcriptomes may overcome this challenge, as it provides more precise expression levels of transcripts, including differentially expressed genes (DEGs) and sequence variations (e.g., single nucleotide polymorphism, SNPs), compared to microarray approaches. The fact that RNA-Seq data reveal a set of early-response genes demonstrates confirmative power for the cell-based assay results, making it an effective and informative approach to elucidate the mechanism of action (MoA) of natural compounds (5). For instance, RNA-Seq has been used to analyze the effects of phenolic acid isolated from *Salvia miltiorrhiza* Bunge (6) and curcumin from *Curcuma longa* (7) on cancer cells. Based on the RNA Seq results, bioinformatics enrichment tools such as Gene Ontology (8), DAVID (9) play a very important role in the gene functional analysis (10). Here, in extending biological insight from our RNA-Seq data of NBT, we used Gene Set Enrichment Analysis (GSEA) (11) to interpret the result of gene expression profiles into biological mechanisms.

In this study, we investigated the detailed biological effects of NBT on cell cycle arrest *in vitro* and *in vivo*. We demonstrated the potential mechanism of NBT inhibiting the release of G1/S and G2/M cell cycle block in HeLa cells was through its effects on the expression of E2F1 and GADD45α, respectively. *In vivo* results further confirmed the tumor inhibitory effect of NBT with no apparent toxicity. Taken together, our results show that NBT is a promising lead compound for further anticancer drug development.

## Materials and Methods

### Chemicals and Reagents

Neobractatin (NBT) with a purity >98% was isolated from *Garcinia bracteata* by Mr. Baojun Zhang and Dr. Wenwei Fu (one of co-authors) in our lab (4). In brief, the air-dried trunks of *Garcinia bracteata* (4.0 kg) was pulverized and extracted with 95% (v/v) ethanol (3 × 8L) at room temperature, filtered and concentrated to give a crude extract (754.8 g). The EtOAc-soluble fraction (262.2 g) was subjected to Si gel column chromatography (Φ10 × 75 cm, 3.0 kg) with a gradient of petroleum ether—acetone as the eluent, and ten fractions (A–J) were collected. Fraction D (14.5 g) was further separated on Si gel, Sephadex LH-20, and RP-C18 Si gel columns to give pure compound Neobractatin. The compound was identified based on MS and NMR spectroscopy analysis and by comparison of their spectroscopic data with published values (4, 12, 13).

### Cell Culture and Synchronization

All cell lines from the cell bank of the Shanghai Institutes of Biochemistry and Cell Biology, Chinese Academy of Sciences were grown in Dulbecco's modified Eagle's medium (Gibco/Invitrogen, 12800-017, Carlsbad, CA, USA) or RPMI 1640 medium (Gibco/Invitrogen, C22400500CP, Carlsbad, CA, USA) as recommended by the providers.

To obtain synchronized cells, HeLa cells were arrested by double thymidine and nocodazole block. The cells were blocked for 18 h with 2 mM thymidine, released for 9 h by washing out the thymidine, and then blocked again with 2 mM thymidine for 17 h to arrest all the cells at the G1/S transition. For the nocodazole release experiments, HeLa cells were blocked with 100 ng/ml nocodazole for 18 h, washed three times in phosphate-buffered saline (PBS), and released into the medium or medium with NBT. The cell cycle distribution was determined by flow cytometry.

### Cell Proliferation Assay

Tumor cell lines were plated in 96 wells. After treatment, 3-(4, 5-dimethylthiazol-2-yl) 2, 5-diphenyltetrazolium bromide (MTT, Sigma) solution was added to the cells and the mixture was further incubated for 4 h at 37°C. After removing the medium, 100 μl DMSO was added and the absorbance was measured at 570 nm using EnSpire 2300 (Perkin Elmer). IBM SPSS Statistics was used to calculate the median inhibitory concentration (IC_50_). The experiments were repeated three times.

The inhibition rate (IR) was determined with following formula: IR (%) = (OD_DMSO_ − OD_NBT_)/OD_DMSO_ × 100%.

### Flow Cytometric Analysis

HeLa cells (2.5 × 10^5^ cells/well) were seeded in 6-well plates. After treatment with NBT or HH, the cells were harvested, washed twice with PBS, and fixed with 70% ethanol in PBS overnight. After being washed with PBS, the cells were then suspended in PBS containing propidium iodide (Sigma, P4170, PI) and RNase (10 μg/mL) for an additional 30 min. Finally, the cells were washed, and the cell cycle distribution was determined using a flow cytometer (FACS Calibur II) equipped with Cell Quest Pro software (BD Biosciences, San Jose, CA, USA); the cytometry results were analyzed using FlowJo software (version VX).

### Western Blot Assay

Cell lysates were prepared in ice-cold whole-cell extract buffer (50 mM TRIS-HCl, pH 8.0, 4 M urea and 1% Triton X-100) supplemented with a complete protease inhibitor mixture (Roche Diagnostics, 04693132001). Aliquots (30 μg) of protein were separated by SDS–polyacrylamide gel electrophoresis (SDS-PAGE) and transferred to polyvinylidene difluoride (PVDF) membranes. After blocking non-specific binding for 1 h (5% non-fat milk in 0.1% TBS/T) at room temperature, the membranes were probed with antibodies as follows: from Abcam, Cambridge, UK: CDK2 (ab32147), GAPDH (ab128915), cyclin E1 (1655-1), p21 (3733-1), c-myc (ab32072), lamin A+C (ab108595); from Cell Signaling Technology, Danvers, MA, USA: cyclin B1 (12231), cyclin D1 (2978), E2F-1 (3742) and phospho-Rb (Ser807/811) (9308); from Santa Cruz Biotechnology, Dallas, TX, USA: cyclin A1 (B-8, sc-271682), and α-tubulin (sc-5286) and GADD45α (A11768, Abclonal Biotechnology), PUMA (55120-1-AP, Proteintech), p53 (bs-8687R, Bioss). After incubation at 4°C overnight, the membranes were incubated with the appropriate secondary antibodies (anti-mouse IgG(H+L) or anti-rabbit IgG(H+L), 1:2,500; SeraCare) for 1 h at room temperature. Protein bands were visualized using an ECL kit (Cat. 54-61-00, KPL). The results were visualized using an ImageQuant LAS 4000 Mini and processed using ImageQuant TL 1D software (General Electric Company).

### Nuclear/Cytoplasmic Fractionation

The Nuclear and Cytoplasmic Protein Extraction Kit (Beyotime, Shanghai, China, #P0027) was used to extract nuclear and cytoplasmic proteins from cultured cells and tissues according to the manufacturer's protocol. Briefly, cells were washed in cold PBS, resuspended in buffer containing 1 mM PMSF, vortexed for 5 s at the highest speed and incubated on ice for 15 min. After the addition of another aliquot of extraction buffer, additional vortexing and placing the sample on ice for 1 min, the nuclei and supernatant (cytoplasm) were separated by centrifugation at 4°C. The nuclei were resuspended in the nuclear extraction buffer, incubated on ice for 30 min, and vortexed at intervals. Nuclear extracts were collected by centrifugation at 14,000 × g for 10 min at 4°C. The cytoplasmic and nuclear fractions were then used for western blotting.

### Immunofluorescence Microscopy

Cells plated on glass coverslips were treated with double thymidine to obtain synchronized cells as described above. After releasing the cells into medium with or without NBT for the indicated periods of time, the cells were fixed in 4% paraformaldehyde (PFA) for 20 min and permeabilized in PBS containing 0.1% Triton X-100 for 10 min at room temperature. Non-specific binding was blocked by incubation of the samples for 30 min in PBS containing 5% BSA and 0.3% Triton X-100. The cells were incubated with primary antibodies against E2F1 (diluted 1:100, KH95, sc-251) overnight at 4°C followed by incubation with Alexa-Fluor 594-conjugated donkey anti-mouse IgG for 1 h at room temperature. 4′6-diamidino-2-phenylindole (DAPI, Invitrogen) staining was then used to stain nuclei. The cells were photographed using an Olympus fluorescence microscope (IX83, Tokyo, Japan).

### Plasmids and Transfection

The plasmid encoding the YFP-tubulin fusion protein was kindly provided by Professor Donald C. Chang of Hong Kong University of Science and Technology. After cultured cells reached 50–80% confluent, plasmids were transfected using Lipofectamine 3000 (Invitrogen) according to the manufacturer's instructions. After 24 h incubation, the cells were synchronized as described before and treated with NBT for DAPI staining. The cells were photographed by confocal microscopy (Leica TCS SP8, 100x oil).

### RNA Isolation and Real-Time PCR Assay

RNA was isolated using Trizol reagent (Beyotime, R0016). One microgram of total RNA was reversed-transcribed using the PrimeScript RT Reagent Kit (TaKaRa, DRR037A). Quantitative PCR was performed with mRNA-specific primers in a StepOnePlus Real-Time PCR System (Applied Biosystems, Life Technologies) using SYBR Green Real-time PCR Master Mix (TOYOBO, QPK-201). The conditions for PCR were one cycle of 10 min at 95°C and 40 cycles of 10 s at 95°C and 30 s at 65°C. The primers for the real-time PCR reactions were as follows:

E2F1 forward, 5′-AGT TCA TCA GCC TTT CCC-3′;
E2F1 reverse, 5′-AGG TCC CCA AAG TCA CAG-3′;
Rb1 forward, 5′-TTA TCA AAG CAG AAG GC AA-3′;
Rb1 reverse, 5′-AGA GGA CAA GCA GAT TCA AG-3′;
cyclin A forward, 5′-CAA TGG ATG GTA GTT TTG AGT-3′;
cyclin A reverse, 5′-GTG ATG TCT GGC TGT TTC TT-3′;
cyclin E forward, 5′-GCC TTG TAT CAT TTC TCG TC-3′;
cyclin E reverse, 5′-GCT GTC TCT GTG GGT CTG-3′;
GADD45α forward, 5′-GAG AGC AGA AGA CCG AA AG-3′;
GADD45α reverse, 5′-GCA GGA TGT TGA TGT CGT-3′
GAPDH forward, 5′- ACG ACC ACT TTG TCA AGC TC-3′;
GAPDH reverse, 5′- GTT GCT GTA GCC AAA TTC GT-3′
18S forward, 5′- GTA ACC CGT TGA ACC CCA TT-3′
18S reverse, 5′- CCA TCC AAT CGG TAG TAG CG-3′

### RNA-Sequencing Analysis

After treatment of cells with NBT (5 μM) for 8 h, total RNA was isolated from the cells using Trizol reagent (Beyotime, R0016) and further purified using the MinElute Cleanup Kit (74204, QIAGEN) and the RNase-Free DNase Set (79254, QIAGEN) according to the manufacturer's instructions. The quality and quantity of the total RNA were assessed using the 2100 Bioanalyzer. Total RNA samples with RNA Integrity Number (RIN) > 8 were used to generate libraries using the TruSeq mRNA Library Kit (Illumina) and sequenced on a HiSeqX PE150 as previously described (14). The entire RNA-seq dataset is available at the Gene Expression Omnibus (GEO) database under the accession number GSE108706. We mapped RNA-seq reads to the transcriptome (Ensembl, release 72) using the software TopHat (v1.4.1) and used Cuffdiff (v2.2.0) to calculate RNA-seq based gene expression levels using the FPKM metric (fragments per kilobase of exon per million fragments mapped). We also used Cuffdiff to identify the genes that were differentially expressed in control and NBT samples using the cutoff of Cuffdiff FDR <5% and an extra filter of *p*-value ≤ 0.01 from a two-sided *t*-test of FPKM values. The normalized expression values are provided in Supplementary Table 1. The enrichment analyses were conducted using the KEGG (http://www.genome.jp/kegg/) and Gene set enrichment analysis (GSEA) (www.broad.mit.edu/gsea) databases as data sources.

### Gene Set Enrichment Analysis (GSEA)

For GSEA analysis, we followed the standard procedure as obtained from the Broad Institute Gene Set Enrichment Analysis website and related references (11). The dataset (.gmt) and phenotype label (.cls) files were created and loaded into GSEA software. The number of permutation was set to 1,000, and the phenotype label was NBT vs. DMSO. The FDR for GSEA is the estimated probability that a gene set with a given NES (normalized enrichment score) represents a false-positive finding, and FDR < 0.25, *p* < 0.05 is considered to be statistically significant for GSEA.

### *In vivo* Animal Model and Immunohistochemistry

This study was carried out in accordance with the recommendations of the Guidelines for the Care and Use of Laboratory Animals, and the protocols were approved by the Shanghai University of Traditional Chinese Medicine Committee on the Use of Live Animals for Teaching and Research (SZY201805008). When the tumor size of the HeLa reached ~50 mm^3^, the mice were randomly divided into four groups for daily intraperitoneal treatment with vehicle control (0.5% DMSO and 5% Tween-80 in normal corn oil), NBT (5 mg/kg and 10 mg/kg in vehicle control), cisplatin (2 mg/kg). The body weights and tumor sizes of all mice were recorded daily. The tumor volumes were measured by Vernier caliper measurement and calculated using the following formula: [(shortest diameter)^2^ × (longest diameter)]/2. After 24 days of administration, all the mice were sacrificed. Explanted tumors were weighed, formalin fixed, and embedded in paraffin for immunohistochemistry.

Tumors were fixed in 4% PFA at room temperature for 48 h. Selected samples were embedded in paraffin, sectioned and stained with Ki-67 (1:200, Abcam, ab16667), GADD45α (1:500, Servicebio, GB11576), E2F1 (1:1,000, Servicebio, GB11571) and cyclin B1 (1:400, Servicebio, GB11255). The sections were mounted for histological analysis and visualized by DAB.

### Statistical Analysis

The data were presented as the mean ± SD. Statistical analysis was performed using 2-tailed Student's *t*-test or one-way ANOVA when more than 2 groups are being evaluated. *P*-values <0.05 were considered to indicate statistically significant differences.

## Results

### NBT Blunts Cell Proliferation and Inhibits the Release of G1/S and G2/M Cell Cycle Block

**Figure 1:**
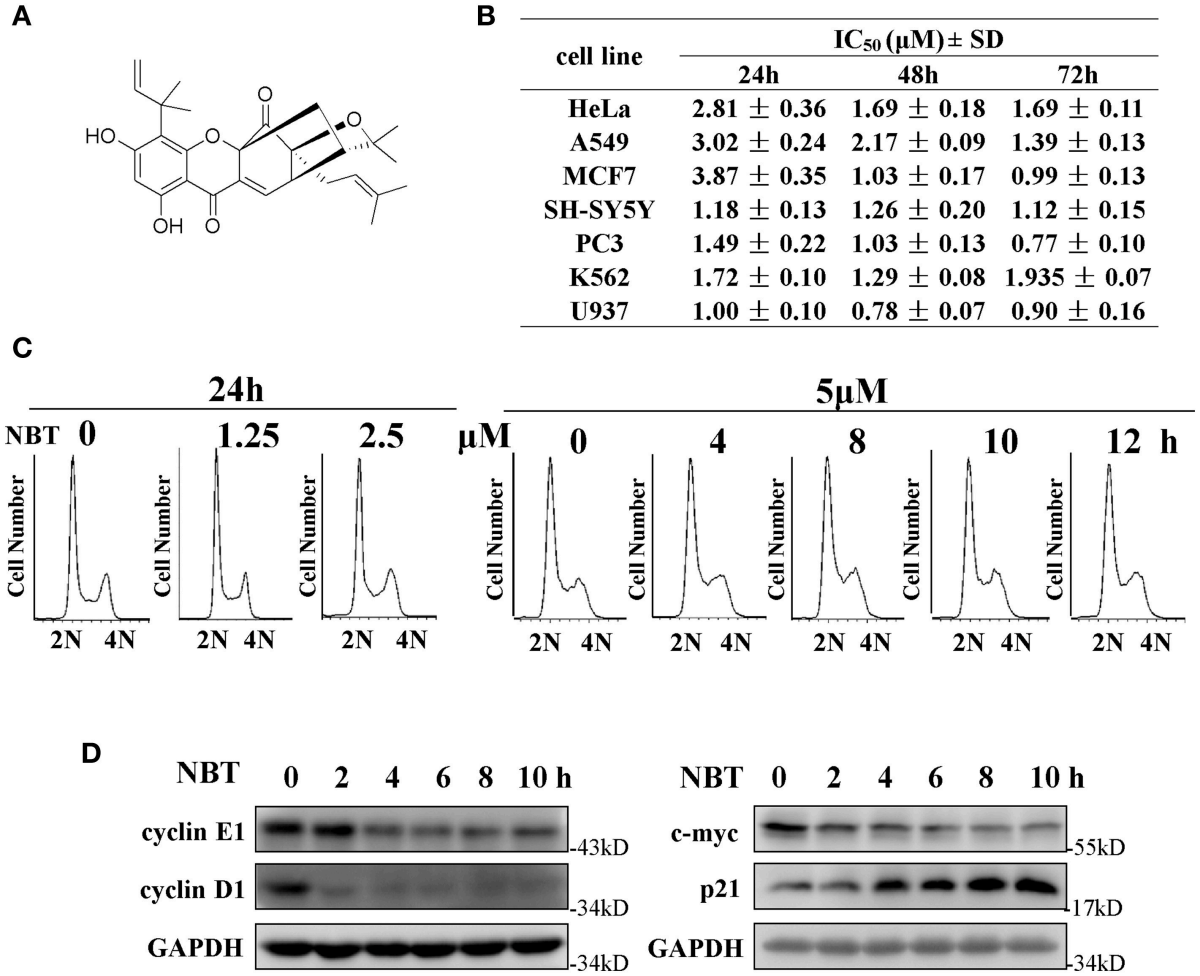
NBT suppresses cell growth and affects cell cycle *in vitro*. **(A)** Chemical structure of NBT. **(B)** Cytotoxicity effects of NBT on seven tumor cell lines. Cells were treated with NBT (0–10 μM) for 24, 48, 72 h. Cell proliferation was measured by the MTT assay. The IC_50_ values are reported as the means ± SD from three independent experiments. **(C)** HeLa cells treated with different concentrations of NBT for 24 h or for different time periods as indicated were analyzed by flow cytometry to determine cell cycle distribution. **(D)** HeLa cells were treated with NBT (5 μM) for varying amounts of time (2, 4, 6, 8, and 10 h), and the cell cycle-related proteins in the samples were analyzed by western blotting (GAPDH as loading control).

We have previously reported that NBT (chemical structure shown in Figure 1A) suppressed the proliferation of several cancer cell lines (4). As shown in Figure 1B, NBT (0–10 μM) treatment exhibited growth inhibitory activity against seven cancer cell lines.

**Figure 2:**
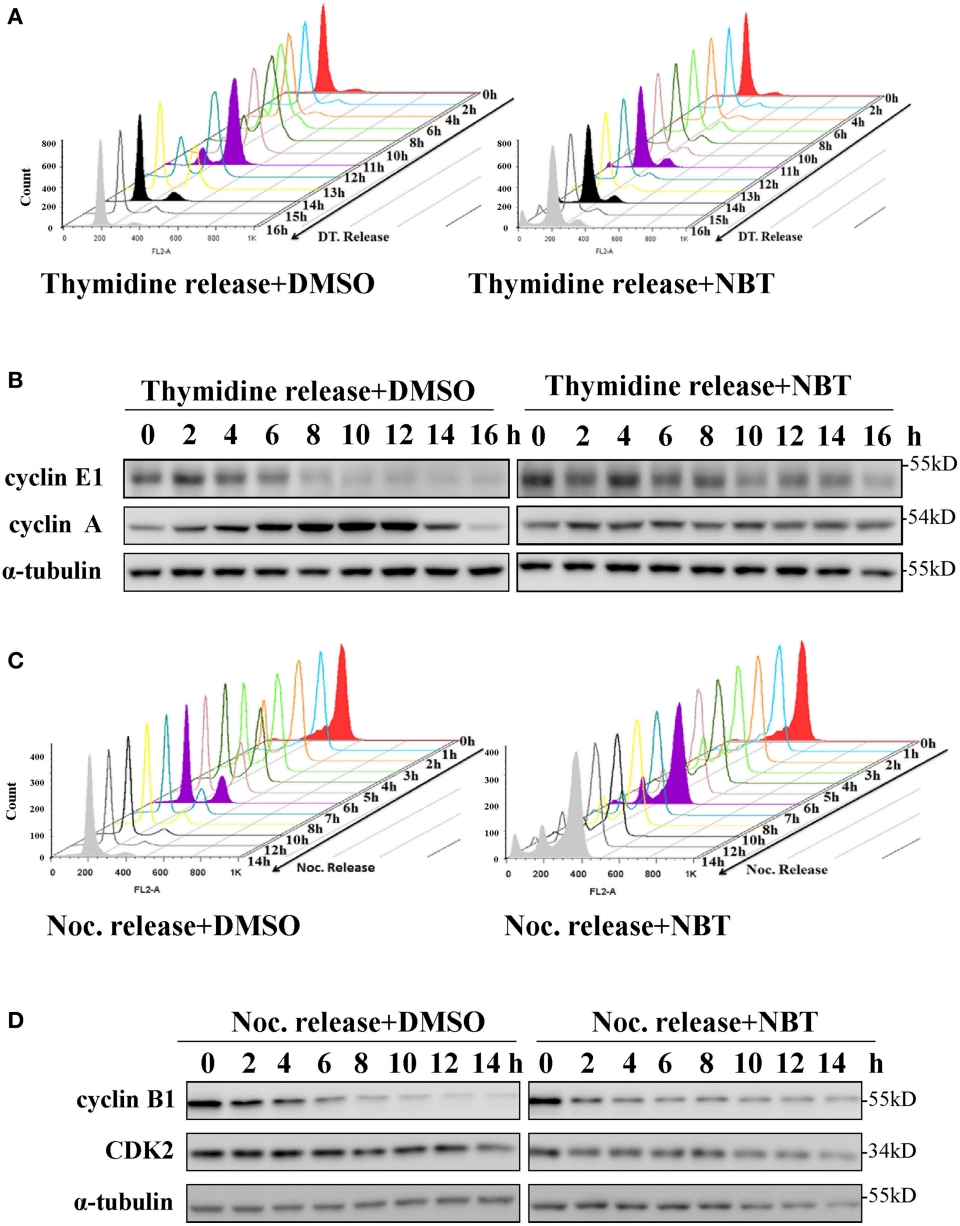
NBT inhibits the release of G1/S and G2/M cell cycle block. HeLa cells were synchronized at G1/S phase using double thymidine treatment (DT, 2 mM) **(A,B)** and at G2/M phase using nocodazole (Noc, 100 ng/ml) **(C,D)** under the treatment of DMSO as control and NBT (5 μM). Cells collected at the indicated time points were analyzed by FACS and western blotting for cyclin E1, cyclin A, cyclin B1, cyclin-dependent kinase 2 (CDK2) and α-tubulin (loading control).

To investigate whether NBT inhibited cancer cell proliferation by inducing cell cycle arrest, we treated the HeLa cell line with NBT and examined the cell cycle distribution by flow cytometry. Surprisingly, treatment with NBT did not result in significant changes in the cell cycle in both time and dose-dependent manner (Figure 1C). However, we observed some key cell cycle-related proteins decreased upon NBT treatment in HeLa cells, including c-myc and some key cyclin proteins, cyclinE1, cyclinD1 for example (Figure 1D). The SYBR Green assay and propidium iodide (PI) uptake were further applied to distinguish the anticancer activity of NBT between cytostatic action and cytotoxicity. The results showed that NBT under the condition of our study exhibited inhibitory growth ability, rather than cell death induction (Supplementary Figures 1A,B). We also investigated the effects of NBT on p53, a well-known tumor suppressor gene in cervical cancer and its downstream proteins. The results suggested that NBT increased levels of p53 and its downstream p21 (cell cycle inhibitor). While puma, a p53-upregulated modulator of the apoptosis gene (15), was found to be only slightly upregulated (Figure 1D; Supplementary Figure 2), which further supported our conclusion that NBT plays a pivotal role in cervical tumor cell growth. To understand this discrepancy and determine whether NBT interferes with progression through the cell cycle, we used two cell-cycle synchronization protocols to carefully evaluate the effect of NBT (16). As shown in Figure 2A, most cells synchronized in G1/S phase after double thymidine treatment were released to normal cell cycle within 16 h (left panel). Upon NBT treatment, the cells were retained in G1/S phase without progression (Figure 2A, right panel). Key cyclins, such as cyclin E1, are normally degraded during cell cycle progression, but upon treatment with NBT, this degradation was delayed. Cyclin A, whose expression levels show dramatic fluctuation during the cell cycle in control cells, remained relatively constant in cells exposed to NBT (Figure 2B). Similarly, when the cells were released from G2/M phase following nocodazole block, NBT also maintained most cells in G2/M phase (Figure 2C). Cyclin-dependent kinase 2 (CDK2) levels showed accelerated decrease after NBT treatment, whereas cyclin B1 protein level was stable after the initial drop within the first 2 h of nocodazole release, in contrast to the sustained decrease seen in DMSO treated cells (Figure 2D). The above data suggest that NBT inhibits G1/S- and G2/M cell cycle release, which explains why there was no apparent difference in the distribution of cells in the cell cycle after NBT treatment (Figure 1C).

### NBT Causes Global Transcriptome Changes

**Figure 3:**
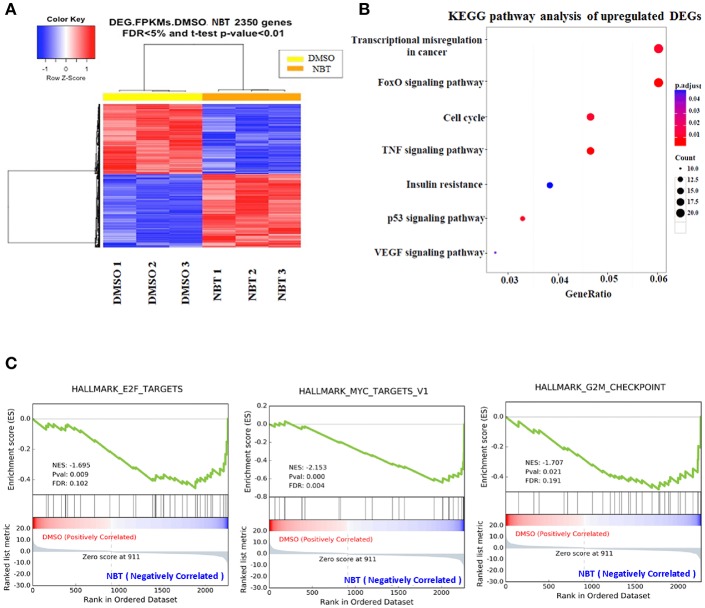
RNA-Seq analysis reveals global transcriptome changes caused by NBT. RNA-Seq was conducted with NBT-treated HeLa cells. **(A)** Heat map of 2,350 differentially expressed genes (DEGs) from RNA-Seq analysis of DMSO and NBT-treated cells. Each row of the heat map represents the Z-score transformed FPKM values of one differentially expressed gene across all samples. **(B)** Kyoto Encyclopedia of Genes and Genomes (KEGG) pathway analysis of upregulated genes in NBT treated cells vs. control conditions. Gene ratio refers to the ratio of the number of target genes associated with a KEGG pathway to the total number of genes in the pathway. The count presented the number of DEGs enriched in a particular pathway. **(C)** Gene expression profile analysis by gene set enrichment analysis (GSEA) revealed a significant enrichment of gene signatures associated with E2F, MYC targets and G2/M checkpoint gene set (*p* < 0.05). Positive (red) and negative (blue) ES indicate enrichment in DMSO-and NBT-treated samples, respectively. Normalized enrichment scores (NES), *p*-value and false discovery rate (FDR) are indicated for each gene set. Y-axes indicate enrichment scores (top) and ranked list metric (bottom). X-axis bars represent individual genes of the indicated gene sets.

In view of the observation that NBT has multiple effects on the cell cycle, we then applied RNA-Seq analysis to further investigate the molecular mechanism underlying NBT action. Analysis of NBT-treated HeLa cells identified 2350 deferentially expressed genes (DEGs) (Supplementary Table 1). In the heat map of the 2350 DEGs induced by NBT, the three DMSO- and three NBT-treated samples were clearly separated by hierarchical clustering (Figure 3A), demonstrating robust transcriptome changes upon NBT treatment. From further analysis of enriched Kyoto Encyclopedia of Genes and Genomes (KEGG) pathways, we found that the most enriched pathways were transcriptional misregulation in cancer and the cell cycle (Figure 3B; Supplementary Table 2), suggesting that NBT caused significant changes in the transcriptome.

To surmise molecular pathways associated with NBT regulated genes, we analyzed our RNA-Seq data with the software tool “Gene Set Enrichment Analysis” (GSEA; www.broad.mit.edu/gsea), which facilitated correlation of gene expression. The genes belonging to a defined pathway were ranked together according to their change in expression in NBT treated cells compared to DMSO treated controls, and a maximum enrichment score (ES) were calculated for each gene set (Supplementary Table 3). As shown in Figure 3C, most of the deregulated gene sets were associated with E2F, MYC targets and G2/M checkpoint. Since the role of E2F/MYC network in regulating the G1/S transition has been well documented (17), our GSEA results suggested that the anticancer effect of NBT was primarily related to both G1/S and G2/M phases, which was consistent with our earlier results of Figure 2.

### NBT Arrests Synchronized Cells in G1/S Phase by Downregulating E2F1 Activity

**Figure 4:**
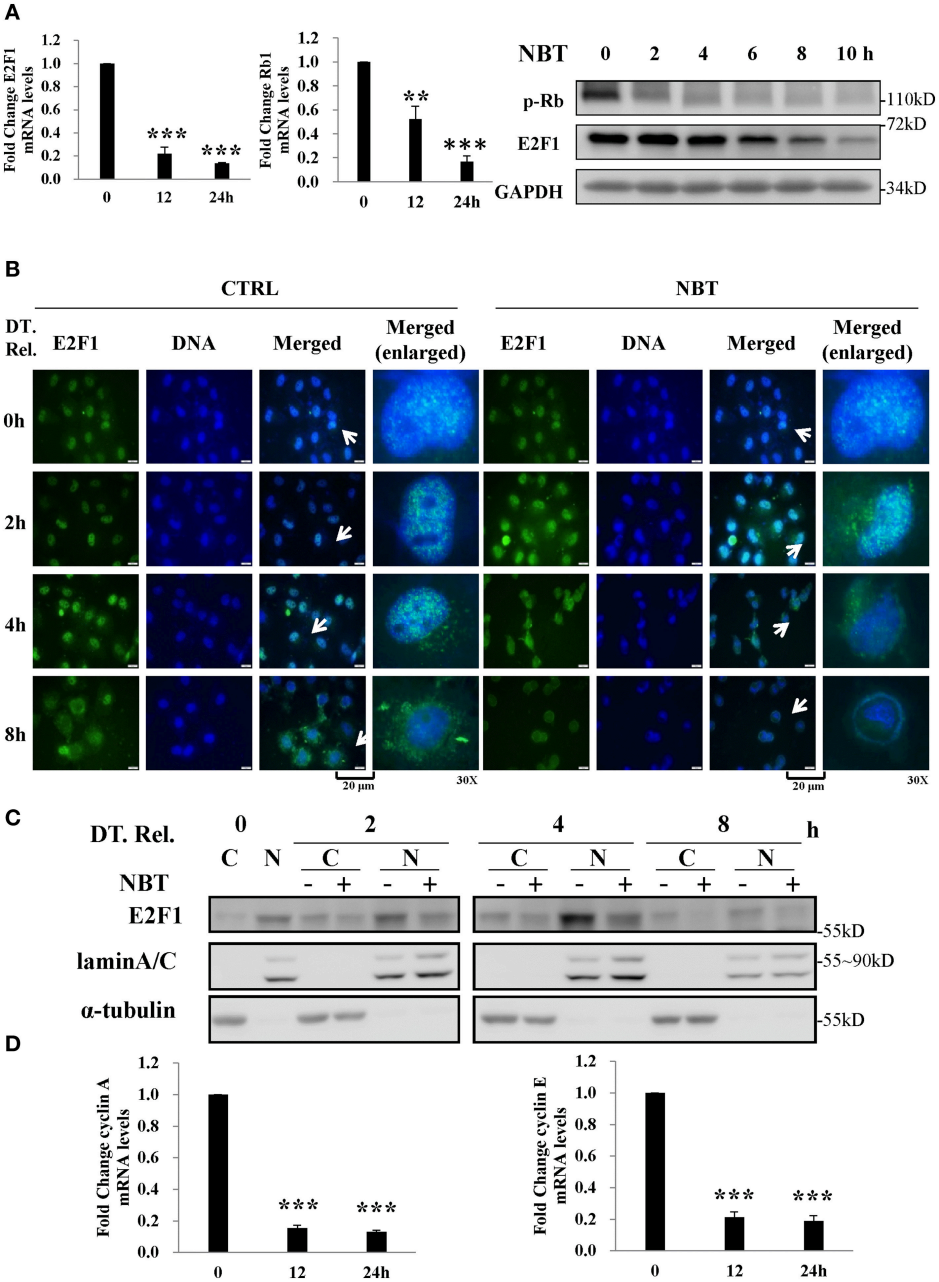
NBT arrests synchronized cells in G1/S phase by decreasing E2F1 activity **(A)** HeLa cells were treated with NBT (5 μM) for 12 or 24 h. Relative E2F1 (left panel) /Rb1 (middle panel) mRNA levels (normalized to 18S mRNA) were analyzed by quantitative RT-PCR. Right panel: HeLa cells were treated with NBT (5 μM) for the indicated time periods (2, 4, 6, 8, and 10 h). Samples were analyzed by western blotting for p-Rb, E2F1, and GAPDH (loading control). **(B,C)** HeLa cells arrested in G1/S phase were released (double thymidine release, DT. Rel.) into fresh medium containing NBT (5 μM) or DMSO. **(B)** Cells were immunostained for E2F1 protein (green) and DAPI (blue) at the indicated times. Scale bars: 20 μm, Magnification: 30X. **(C)** Western blot analysis was performed after the preparation of nuclear (N) and cytosolic **(C)** fractions. **(D)** HeLa cells were treated with NBT (5 μM) for 12 or 24 h. Relative cyclin A and cyclin E mRNA levels (normalized to 18S) were analyzed by quantitative RT-PCR. The data shown are the means ± SD from 2 independent experiments and were analyzed using ANOVA (**P < 0.01, ***P < 0.001).

Based on our GSEA analysis, deregulated gene sets related to G1/S phase were associated with E2F targets (Figure 3C), we detected several members of E2F family and found that E2F1 was significantly affected by NBT (data not shown).As a repressor of E2F/DP heterodimeric transcription factor 1 (E2F1) target genes, the retinoblastoma 1 (Rb1) is known to interact with it, inhibiting its transcriptional activity, thus preventing entry into S phase and causing G1 arrest of cycling cells (18, 19). To test whether the Rb1 and E2F1 complexes were involved in the NBT-mediated G1/S cell cycle arrest, we treated HeLa cells with NBT and found a significant decrease in E2F1and Rb1 mRNA levels as well as declined E2F1 and phosphorylated Rb1 protein levels, compared to untreated cells (Figure 4A). This finding suggests that NBT downregulates E2F1 and phosphorylated Rb1, which consequently block the G1-to-S transition.

Alterations in the localization of the transcription factor E2F1 have been shown to play a vital role in its transcriptional activity (20, 21). To examine whether NBT treatment affects E2F1 translocation, we examined the localization of E2F1 in double thymidine-synchronized cells after NBT treatment. As shown in Figure 4B, NBT modulated the subcellular localization of E2F1 within 2 h, compared to 4 h in cells treated with DMSO. Western blotting results also showed that the amount of E2F1 in the nuclear fraction decreased upon NBT treatment compared with that in cells treated with DMSO, which possibly due to translocation of E2F1 or decrease in total protein (Figure 4C). Correspondingly, NBT treatment led to a reduction in the mRNA levels of cyclin A and cyclin E (Figure 4D), 2 well-known gene targets of E2F.

Taken together, our results show that NBT inhibits E2F1 transcription and affects the Rb–E2F pathway, which in turn result in G1/S arrest.

### NBT Causes G2/M Arrest Through Upregulation of GADD45α and the Disruption of Mitotic Spindle Formation

**Figure 5:**
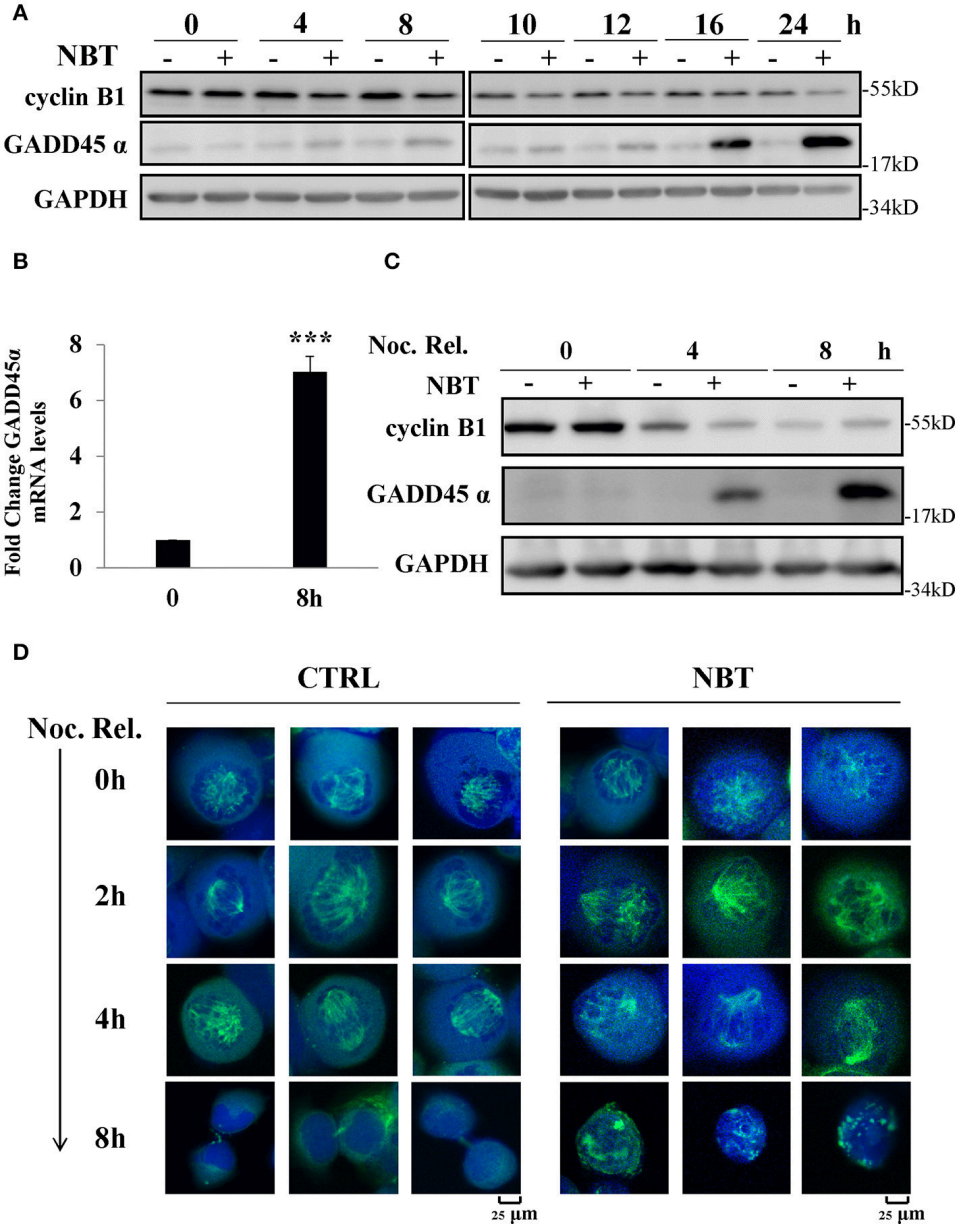
NBT causes G2/M arrest through up-regulation of GADD45α and the disruption of mitotic spindle formation. **(A)** Western blot analysis of mitosis-related proteins (cyclin B1 and GADD45α, GAPDH as loading control) after treatment of HeLa cells with 5 μM NBT. **(B)** Cells were treated with NBT (5 μM) for 8 h. Relative GADD45α mRNA levels (normalized to 18S mRNA) were analyzed by quantitative RT-PCR. The data shown are the means ± SD from 3 independent experiments and were analyzed using Student's *t*-test (***p < 0.001). **(C)** HeLa cells pre-treated with nocodazole (100 ng/ml) for 18 h were released into fresh medium or into medium containing NBT at the indicated times. Samples were analyzed by western blotting. **(D)** HeLa cells transfected with YFP-tubulin (green) were treated with nocodazole as in **(C)**, Cells were stained with DAPI (blue) and analyzed by confocal microscopy (Leica TCS SP8, 100x oil). Scale bars: 25 μm. Noc. Rel. (nocodazole release).

We next investigated the possible mechanisms of NBT causing G2/M arrest. Growth arrest and DNA-damage-inducible protein, GADD45α, and cyclin B1 have been reported to play essential roles during early mitosis of cell (22). The gene expression of GADD45α was also noticeably increased according to our Seq data (Supplementary Table 1). Western blot analysis showed changes in cyclin B1 and GADD45α protein when treating unsynchronized (Figure 5A) and synchronized (Figure 5C) HeLa cells with NBT. We found that NBT treatment caused a significant increment in GADD45α protein levels and decrement in the mitotic cyclin B1 protein level in a time-dependent manner (Figure 5A). Concordantly, the mRNA levels of GADD45α were also significantly upregulated (Figure 5B). In addition, in the mitotically synchronized of NBT-treated cells, GADD45α protein expression increased at a very early time point (4 h) (Figure 5C) compared to that in unsynchronized cells (16 h) (Figure 5A). Cyclin B1 exhibited a steep reduction within 4 h, but then stabilized at 8 h, in contrast to a continuous decrease over time when treated with DMSO (Figure 5C). These data suggest that NBT induces the alteration of GADD45α and cyclin B1, which at least partially contribute to G2/M arrest.

Since GADD45α is related to microtubule stability (23), and mitotic spindle assembly using microtubule is crucial for cells at mitosis, we examined whether NBT affected the formation or stability of the mitotic spindle as visualized by ectopic expression of YFP-tubulin in mitotically arrested cells. Figure 5D showed the structure of the microtubule network in representative cells treated with NBT. While the microtubule organization within the spindle remained intact in NBT-treated cells, the mitotic spindles themselves were kept from renormalizing.

In conclusion, these observations suggest that NBT treatment results in the upregulation of GADD45α, deregulation of cyclin B1 and the disruption of the mitotic spindle, which then causes G2/M cell cycle arrest.

### NBT Hinders Tumor Progression *in vivo*

**Figure 6:**
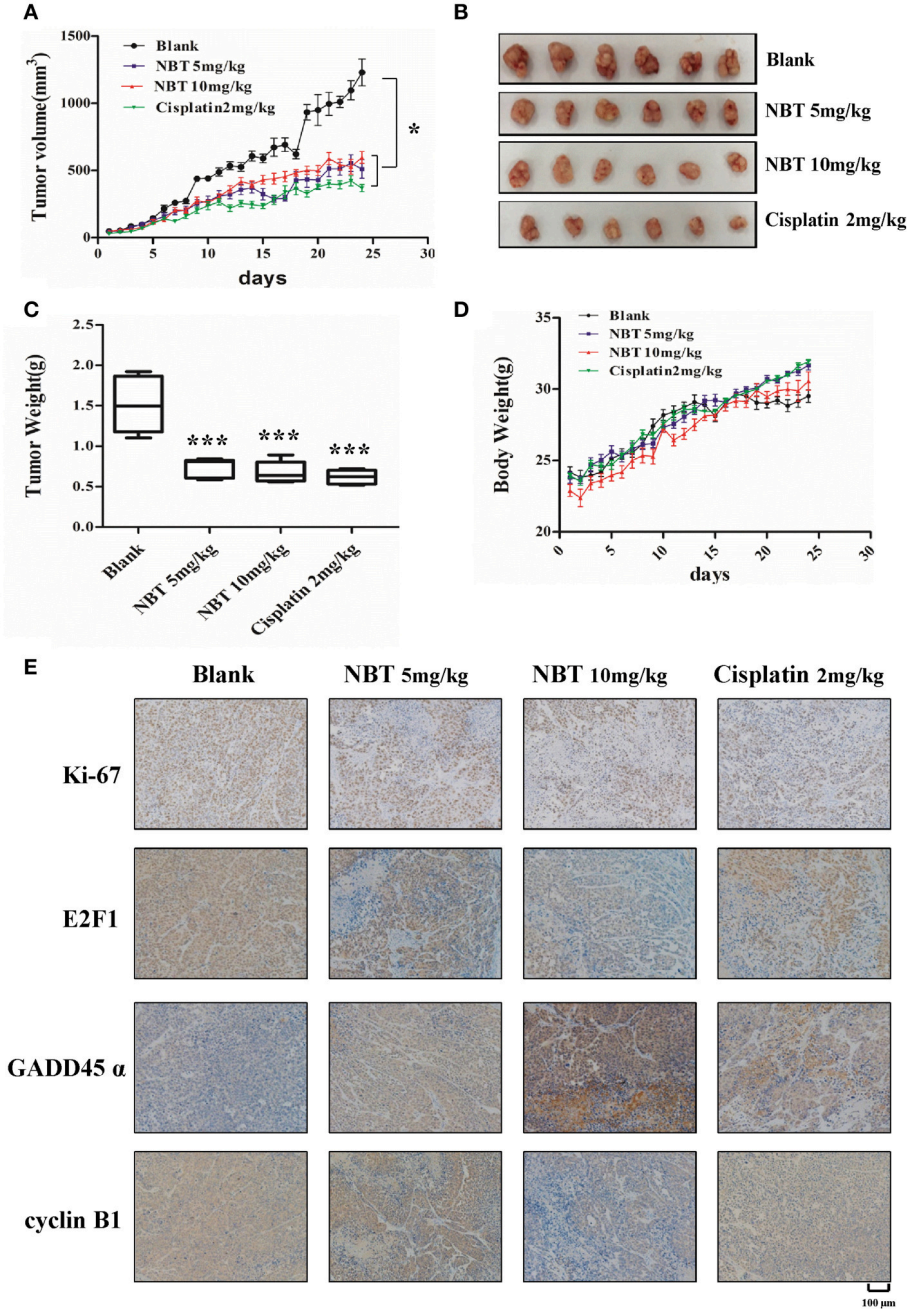
NBT hinders tumor progression *in vivo*. Five-week-old nude mice were engrafted with HeLa cells and randomly divided into four groups. The tumor-bearing mice were then treated with blank, cisplatin (2 mg/kg), NBT (5 mg/kg) or NBT (10 mg/kg) by intraperitoneal injection once daily for 24 days. Tumor volumes were calculated after measuring the length and width of the tumors daily using Vernier calipers. Tumor volume **(A)** and body weight **(D)** were measured daily. **(B)** Representative picture showing tumors excised from different treatment groups. **(C)** Tumor weights are represented as means of tumor weights ± SD. Statistical significance between groups was determined using ANOVA (*P < 0.05, ***P < 0.001). **(E)** Histopathology of xenograft tumors. The tumor sections were treated with immunohistochemical (IHC) staining for Ki-67, E2F1, GADD45α and cyclin B1. The nucleus and cytoplasm brown staining represented positive labeling for the antibody. Representative tumor sections were shown. Scale bars: 100 μm.

To establish the relative contribution of NBT to cancer growth and development *in vivo*, we initially assessed tumor formation using HeLa xenograft model. The effect of daily intraperitoneal administration of cisplatin (2 mg/kg), NBT (5 mg/kg and 10 mg/kg) and vehicle on tumor growth was monitored by measuring tumor size daily. Here, we used cisplatin as a positive control. The tumor size curves and images showed a significant reduction of tumor burden in mice after NBT treatments, with more than 50% decrease vs. the control group. There was no significant difference between the mean tumor volumes of the cisplatin and NBT treated groups (Figures 6A,B). Consistent with the reduction in tumor growth, NBT-treated mice also showed a decrease in tumor weight (Figure 6C). Importantly, we observed no significant change in mouse behavior or loss in body weight (Figure 6D), indicating that the NBT treatments did not result in major toxicity.

To further elucidate the molecular mechanisms of NBT *in vivo*, we used immunohistochemistry (IHC) to measure the levels of the proliferation marker Ki67, E2F1, GADD45α and cyclin B1, as have been detected in our *in vitro* study. We found reduced Ki67 levels in NBT treated tumors, indicating decreased tumor proliferation *in vivo*. Moreover, the expression of E2F1 was greatly decreased. And for the G2/M related proteins, we observed highly increased expression of GADD45α and declined level of cyclin B1 in the tumors after NBT administration (Figure 6E; Supplementary Figure 3), suggesting NBT could cause G2/M arrest *in vivo*, all of which were in agreement with our *in vitro* results. Together, results from *in vivo* HeLa xenograft model studies confirmed our *in vitro* cell line studies and demonstrated the anti-proliferative and cell cycle arrest effects of NBT.

## Discussion

NBT has been shown to regulate apoptosis, autophagy and cell proliferation (4). Although the exact MoA of NBT is still unclear, we have achieved progress in understanding its effect on the cell cycle and cell proliferation. In the present study, we employed RNA-Seq and transcriptome analysis to evaluate the biological effects of NBT. We found that NBT caused arrest in both G1/S and G2/M synchronized cancer cells by regulating the expression of E2F1 and GADD45α respectively. The *in vivo* data further confirmed and demonstrated the potential anti-cancer effect of NBT.

The genome sequencing allows the biological functions to be investigated in a comprehensive, unbiased and hypothesis-free manner (24). Given the advantages of various RNA-Seq analysis tools, it is possible to develop an integrative analysis based on the latest bioinformatics and deep-learning methods to predict potential MoA and clinical applications of natural compounds, herbal extracts, or even herbal decoctions, which may contain mixtures of active compounds. Here we showed that the application of RNA-Seq and transcriptome analysis provided a rapid and efficient method to elucidate the complex biological effects of a novel natural compound. The results of RNA-Seq analysis indicate that several important signaling pathways, including transcriptome misregulation, FoxO signaling pathway, and the cell cycle, may be potentially involved in the MoA of NBT. FoxO is well-studied for its critical role in cancer cell fate, including cell proliferation (25), tumor suppression, longevity, development, and metabolism (26). Since some studies have been reported that FoxO family is involved in autophagy regulation (27, 28), and considering our previous study of NBT in autophagy flux, the effect of NBT on FoxO needs to be further investigated. In this study, we mainly focused on the effect of NBT on cell cycle.

GSEA analysis techniques assist to decipher the long list of gene expression data into more easily interpretable biological pathways. In our GSEA screening, the targets associated with cell cycle were enriched in E2F, MYC and G2/M checkpoint (Figure 3C). E2F are known to regulate the transition of G1 to S phase in cell cycle, as well as governing apoptosis and differentiation (29). The functional properties of E2F family members are closely connected with those of MYC. Many of the early events of the cell cycle regulated by E2F transcription factor are also regulated by MYC (30). Furthermore, the action of MYC in S phase induction is mediated by E2F activity (31). Our study highlights the role of E2F1 and GADD45α in NBT mediated cell cycle arrest. We identified that NBT significantly decreased the expression of E2F1, and possibly affect its translocation. We also observed that NBT downregulated pRb in both mRNA and protein levels. More studies were required to elucidate the effect of NBT on the transcriptional activity of E2F1 and its binding with the members of the retinoblastoma tumor suppressor protein family (pRb, p107, and p130). GADD45α proteins have various roles in the regulation of cell growth including as one of the critical components in regulating cell cycle G2/M arrest (32). With regard to the molecular mechanism by which NBT regulated GADD45α function at the G2/M checkpoint, we showed NBT increased both mRNA and protein expression levels of GADD45α, which might further lead to the destruction of microtubule stability. In further support of these findings, the detail mechanism on how NBT regulated GADD45α to destabilize microtubule, thus causing G2/M arrest was to be investigated. As a critical regulator of cytoskeletal structure, EF-1α was reported to physically interact with increased GADD45α to impair cytoskeletal stability (23). Additionally, the nuclear translocation of GADD45α correlates with the activity of Cdc2-cyclin B1 complex, which also regulates cell cycle G2/M checkpoint (33). We therefore would further focus on detecting the function of NBT on both nuclear localization signal of GADD45α and the binding with EF-1α in our future study.

## Data Availability

The entire RNA-seq dataset is available at the Gene Expression Omnibus (GEO) database under the accession number GSE108706 https://www.ncbi.nlm.nih.gov/geo/query/acc.cgi?acc=GSE108706.

## Ethics Statement

This study was carried out in accordance with the recommendations of the Guidelines for the Care and Use of Laboratory Animals, and the protocols were approved by the Shanghai University of Traditional Chinese Medicine Committee on the Use of Live Animals for Teaching and Research (SZY201805008).

## Author Contributions

YL, LL, HX, and ZZ designed the whole experimental process. ZZ and MW acquired and analyzed most of the data. NX, JZ, and WF gave technical or material support. LL, ZZ, and MW written and reviewed the manuscript. YL, LL, and HX supervised the study. All authors read and approved the final manuscript.

### Conflict of Interest Statement

The authors declare that the research was conducted in the absence of any commercial or financial relationships that could be construed as a potential conflict of interest.

## Supplementary Material

Supplementary Table 1 Expression analysis of deferentially expressed genes (DEGs). The FPKM value of HeLa cells treated with NBT (5 μM for 8 h) identified 2350 DEGs in the cells with RNA-Seq analysis.

Supplementary Table 2 Kyoto Encyclopedia of Genes and Genomes (KEGG) pathways analysis of upregulated DEGs. GeneRatio value of upregulated DEGs for each KEGG pathway description.

Supplementary Table 3 Gene expression analysis by gene set enrichment analysis (GSEA). Columns indicate the name of the gene set. Tables are sorted by increasing FDR. level.

Supplementary Figure 1 NBT inhibited DNA synthesis in HeLa cells. (A) NBT at indicated concentrations was introduced at the time of induction and the cells were harvested and assessed for DNA content using the SYBR Green assay at 12, 24, 48 and 72 h. Data are shown as the mean ± S.D. ^**^*P* < 0.01, ^***^*P* < 0.001 vs. control. (B) Flow cytometry of Propidium Iodide staining was used to detect living cells with or without NBT (5μM) at indicated times (2,4,6,8,12h).

Supplementary Figure 2 NBT increased p53 expression. HeLa cells were treated with NBT (5μM) for varying amounts of time (2, 4, 6, 8 and 10 h), p53and puma were analyzed by western blotting. (GAPDH as loading control).

Supplementary Figure 3 The quantitative analysis of E2F1 and GADD45 a *in vivo*. E2F1 and GADD45 a protein expression levels were analyzed by calculating the integrated IOD/area using Image-Pro Plus version 6.0. IOD/area: Integrated optical density per stained area. ^**^*P* < 0.01, ^***^*P* < 0.001 compared with control group using Student's t test.
