# Supplementary material for: The Natural Compound Neobractatin Induces Cell Cycle Arrest by Regulating E2F1 and Gadd45α
Source: Front Oncol. 2019 Jul 17;9:654. doi: 10.3389/fonc.2019.00654 (PMC6653061; doi:10.3389/fonc.2019.00654)
Supplement: Supplementary Figure 1 — NBT inhibited DNA synthesis in HeLa cells. (A) NBT at indicated concentrations was introduced at the time of induction and the cells were harvested and assessed for DNA content using the SYBR Green assay at 12, 24, 48 and 72 h. Data are shown as the mean ± S.D. **P < 0.01, ***P < 0.001 vs. control. (B) Flow cytometry of Propidium Iodide staining was used to detect living cells with or without NBT (5μM) at indicated times (2,4,6,8,12h). [file Data_Sheet_1.PDF]

# *Supplementary Material*

## 1 Supplementary Methods

### SYBR Green assay

Hela cells (8000 cells/well) were seeded in 96-well plates and cultured with complete medium at various concentrations of NBT. The same number of cells were kept as a baseline and stored at −80 °C. After treatment for 12, 24, 48h, the medium was aspirated and 100 μl of lysis buffer containing SYBR Green at 1:10 000 dilution was added. The cells were lysed in the dark for 1.5 h. The fluorescence intensity of SYBR Green-stained DNA was measured with excitation at 485/20 nm and emission at 528/20 nm using EnSpire 2300 (Perkin Elmer).

### Propidium Iodide Uptake

Hela cells ($2.5 \times 10^5$ cells/well) were seeded in a six-well plate and cultured with complete medium containing DMSO or NBT. Cells harvested at the indicated times were incubated in 5 μg/ml Propidium Iodide in PBS. Cell death was quantitated using a flow cytometer (FACSCalibur II) equipped with CellQuest Pro software (BD Biosciences, San Jose, CA, USA), and analyzed using FlowJo software (version VX).

## 2 Supplementary Figures and legends

### Figure S1

**A**

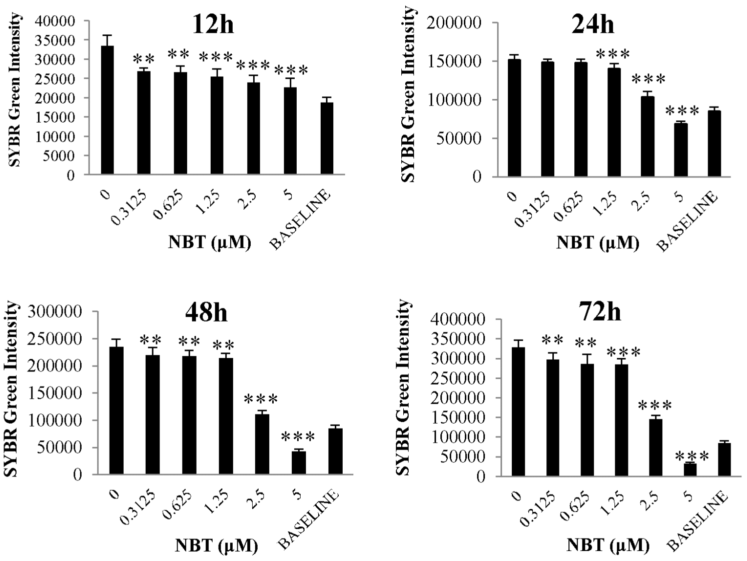


**B**

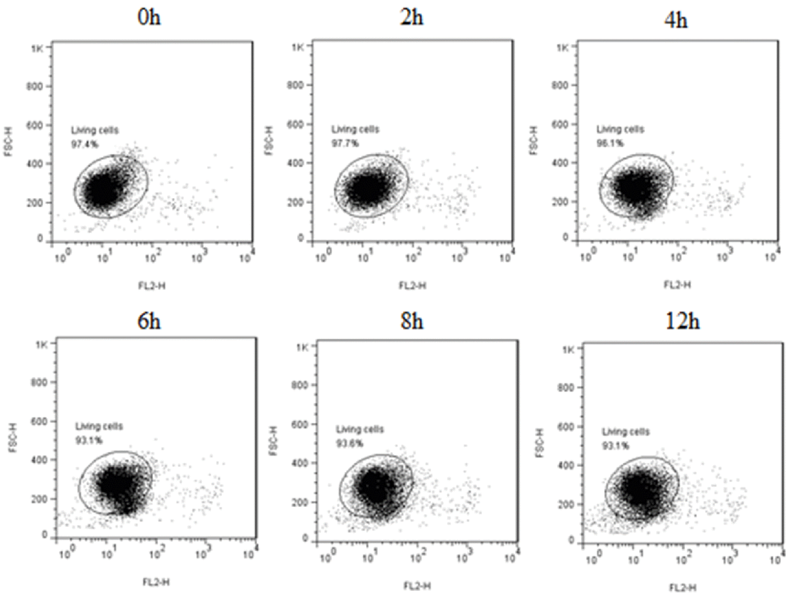


**Supplementary Figure 1. NBT inhibited DNA synthesis in HeLa cells.**

**(A)** NBT at indicated concentrations was introduced at the time of induction and the cells were harvested and assessed for DNA content using the SYBR Green assay at 12, 24, 48 and 72 h. Data are shown as the mean ± S.D. **P < 0.01; ***P < 0.001 versus control. **(B)** Flow cytometry of Propidium Iodide staining was used to detect living cells with or without NBT (5μM) at indicated times (2,4,6,8,12h).

**Figure S2**

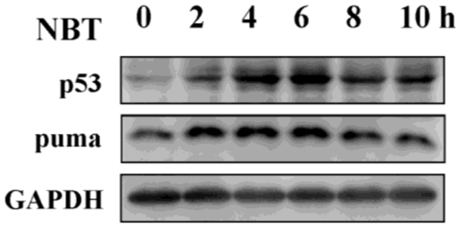


**Supplementary Figure 2. NBT increased p53 expression.**

HeLa cells were treated with NBT (5 μM) for varying amounts of time (2, 4, 6, 8 and 10 h), p53and puma were analyzed by western blotting. (GAPDH as loading control).

**Figure S3**

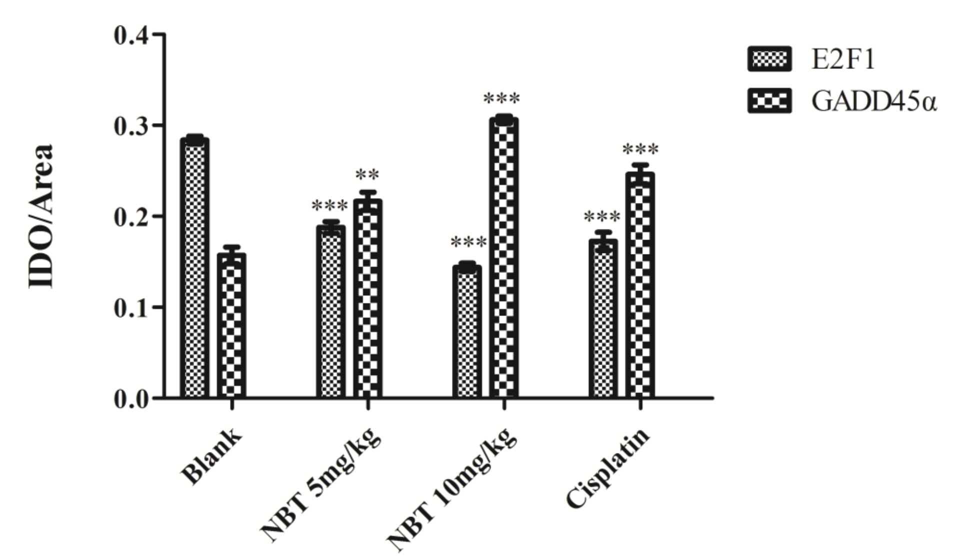


**Supplementary Figure 3. The quantitative analysis of E2F1 and GADD45 α *in vivo*.**

E2F1 and GADD45 α protein expression levels were analyzed by calculating the integrated IOD/area using Image-Pro Plus version 6.0. IOD/area: Integrated optical density per stained area. **P < 0.01; ***P < 0.001 compared with control group using Student's t test.
